# Application of Raman spectroscopy for detection of histologically distinct areas in formalin-fixed paraffin-embedded glioblastoma

**DOI:** 10.1093/noajnl/vdab077

**Published:** 2021-06-18

**Authors:** Gilbert Georg Klamminger, Jean-Jacques Gérardy, Finn Jelke, Giulia Mirizzi, Rédouane Slimani, Karoline Klein, Andreas Husch, Frank Hertel, Michel Mittelbronn, Felix B Kleine-Borgmann

**Affiliations:** 1 Saarland University Medical Center and Faculty of Medicine, Homburg, Germany; 2 National Center of Pathology (NCP), Laboratoire national de santé (LNS), Dudelange, Luxembourg; 3 Luxembourg Center of Neuropathology (LCNP), Dudelange, Luxembourg; 4 National Center of Neurosurgery, Centre Hospitalier de Luxembourg (CHL), Luxembourg, Luxembourg; 5 Department of Oncology (DONC), Luxembourg Institute of Health (LIH), Luxembourg, Luxembourg; 6 Doctoral School in Science and Engineering (DSSE), University of Luxembourg (UL), Esch-sur-Alzette, Luxembourg; 7 Luxembourg Centre of Systems Biomedicine (LCSB), University of Luxembourg (UL), Esch-sur-Alzette, Luxembourg

**Keywords:** FFPE, glioblastoma, machine learning, pathology, Raman spectroscopy

## Abstract

**Background:**

Although microscopic assessment is still the diagnostic gold standard in pathology, non-light microscopic methods such as new imaging methods and molecular pathology have considerably contributed to more precise diagnostics. As an upcoming method, Raman spectroscopy (RS) offers a “molecular fingerprint” that could be used to differentiate tissue heterogeneity or diagnostic entities. RS has been successfully applied on fresh and frozen tissue, however more aggressively, chemically treated tissue such as formalin-fixed, paraffin-embedded (FFPE) samples are challenging for RS.

**Methods:**

To address this issue, we examined FFPE samples of morphologically highly heterogeneous glioblastoma (GBM) using RS in order to classify histologically defined GBM areas according to RS spectral properties. We have set up an SVM (support vector machine)-based classifier in a training cohort and corroborated our findings in a validation cohort.

**Results:**

Our trained classifier identified distinct histological areas such as tumor core and necroses in GBM with an overall accuracy of 70.5% based on the spectral properties of RS. With an absolute misclassification of 21 out of 471 Raman measurements, our classifier has the property of precisely distinguishing between normal-appearing brain tissue and necrosis. When verifying the suitability of our classifier system in a second independent dataset, very little overlap between necrosis and normal-appearing brain tissue can be detected.

**Conclusion:**

These findings show that histologically highly variable samples such as GBM can be reliably recognized by their spectral properties using RS. As conclusion, we propose that RS may serve useful as a future method in the pathological toolbox.

**Key Points**

- Establishment of a SVM (support vector machine)-based classifier to distinguish distinct histological areas in a heterogenous tumor.
- Applicable use of Raman spectroscopy on chemically treated FFPE tissue during the routine pathological workflow.

**Importance of the Study**

In this study, we established an SVM (support vector machine)-based classifier, which can identify specific histological areas in glioblastoma by the use of spectral tissue properties. In contrast to other studies, our classifier can be used on aggressively chemically treated formalin-fixed paraffin-embedded tissue and also on small tissue fragments. Due to its unbiased approach to tumor diagnostics with very little requirements to the sample, Raman Spectroscopy may be a useful, relatively cheap and easy-to-apply future tool in the toolbox of pathology departments. In addition, we have shown that a heterogeneous tissue cannot be determined by a single spectral property. This must be taken into account when creating a global RS-based classifier to differentiate between several entities.

Spectroscopic techniques such as Raman spectroscopy recently gained more attention in tumor research.^1–3^ The Raman effect is the process of inelastic scattering of photons, first described in 1928 by the Indian physicist, C.V. Raman.^4^ The resulting possible differentiation of tissue types opens up opportunities for its diagnostic use in surgery and pathology.^1,5,6^ Therefore, Raman spectroscopy is investigated as both an intraoperative tool due to its non-invasiveness and short integration time,^6^ and for its use on processed tissue. The formalin fixation process causes, however, severe changes of the spectral properties that need to be taken into account when comparing fresh and processed tissue.^7,8^

We went one step further and performed RS on formalin-fixed, paraffin-embedded samples additionally offering precise histological information on adjacent stained serial sections. The general suitability of FFPE tissue for RS was demonstrated already on the small intestine of rats^9^ as well as for the distinction of different tumor entities,^10^ albeit without a focus on tissue heterogeneity. Changes in the Raman Spectra due to the influence of bound paraffin wax (certain points at 1063, 1133, 1296, and 1441 cm^–1^ in the Raman spectra can be assigned to contributing paraffin^11^) make the analysis of the data more complex, even after an efficient dewaxing agent was applied.^11,12^ Furthermore, the choice of slide substrate is crucial due to the small thickness of the sections; while silicon-based glass has a strong Raman signal, CaF_2_ slides (Supplementary Figure 1) with only one single peak at 321cm^–1^, are suitable for Raman Spectroscopy on thin sections.^13^ While these obstacles make the conditions for spectroscopic examinations difficult, the possibility to obtain the precise orientation of the sample as well as the identification of certain areas, and therefore the possible option of accessing even a heterogenous tissue fragment (eg, heterogenous tumor entity) precisely, make this effort worthwhile.

Due to its high heterogeneity, which also reflects its previously used name “glioblastoma multiforme,” we chose glioblastoma (GBM) tumor tissue to analyze the suitability of classifying histological tumor areas using RS on FFPE tissue. GBMs are traditionally characterized and diagnosed according to histopathological features and more recently also genetic alterations.^14^ Amongst multiple highly variable histological features, GBM usually show a vital, a necrotic, and an infiltrative peritumoral zone.^14–17^ Apart from a traditional assessment of the histology in the light microscopic examination, new techniques and artificial intelligence can in the meanwhile diagnose GBM with a similar or even higher precision than experienced neuropathologists.^18–21^

For this reason, data usually needs to be processed and classified by machine learning^22,23^ algorithms, which have strongly influenced the pathology workflow in recent years.^24–26^ Machine learning tools, as employed in this study, are additionally used for prediction of large vessel occlusion (LVO) stroke^27^ and categorization of traumatological patients and prediction of bladder rupture.^28^

This present study was designed to examine Raman spectra of different histopathological areas of GBM and to differentiate them with a MATLAB based classifier that we developed in order to test general suitability of RS as a further diagnostic tool in neuropathology.

## Materials and Methods

### Patient Data

One hundred and seventeen FFPE tissue blocks from 59 tumors of 53 GBM patients were included in the study. For a more detailed description of the glioblastomas, see Supplementary Table 1. The patients underwent routine diagnostics at the National Center of Pathology (NCP) at the Laboratoire national de santé (LNS, Luxembourg) in the years 2018–2020 and were all part of the INSITU® study. The pathological diagnosis for each tumor was carried out by a board-certified neuropathologist (MM) based on histology, immunohistochemistry as well as on epigenetic and genetic results. Histological tumor areas that were analyzed in the present study were defined by means of light microscopic examination. The present INSITU® study, Nr. 201804/08, has been approved by the “*Comité National d”Ethique de Recherche*’ (CNER). It is handled according to the “*EU General Data Protection Regulation* GDPR,^29^ as well as the world medical declaration (WMA) Declaration of Helsinki as a statement of ethical principles for medical research involving human subjects.^30^

### Tissue Processing and Slide Preparation

**Figure 1.**
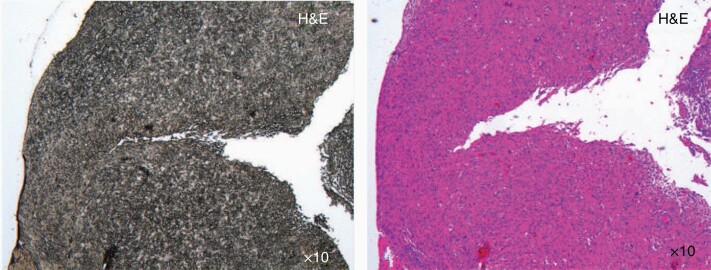
Correlation of Raman spectroscopy and classic histology. (A) Unstained tissue on a CaF_2_ slide. (B) Colored tissue (HE) on a glass slide. Histologically distinct tissue areas were defined in HE samples (here: example of the vital tumor zone) and visually correlated on subsequent serial CaF_2_ slides.

The FFPE tissue blocks were cut with a microtome (thickness of 7μm was chosen, in order to receive a sufficient amount of tissue as well as the possibility of a light microscopical examination) and two consecutive sections were put on two slides. The first section was placed on a glass slide and then HE (hematoxylin and eosin) stained for histological examination, the second section was placed on a CaF_2_ (calcium fluoride; Crystran, Poole, UK) slide and left unstained to prevent spectral contamination. With a single peak^31^ at 321 cm^–1^ (Supplementary Figure 1), CaF_2_ slides have spectral properties that allow proper spectroscopic examination of the tissue on top. In order to reduce the impact of paraffin on the spectra, the slides were dewaxed. The slides were first heated for 60 min at 60°C and then placed in a bath of xylene for 2 × 15 min before being put in descending ethanol solutions for 3 × 2 min. In the next step, distinct areas of GBM (vital GBM core/necrosis/peritumoral zone) were defined on HE stained slides. For easier optical handling, distinct areas were marked by circling them with a marking pen on the backside, before being matched and correlated with the corresponding areas on the CaF_2_ slide (Figure 1). The vital zone was defined as an area with increased cell density, nuclear pleomorphisms, increased mitotic rate as well as facultative hypervascularization. The necrosis zone was defined by eosinophilic areas within the tumor in which nuclei did not stain with hematoxylin anymore. The peritumoral zone was defined by only very mildly elevated cell density, rather containing reactive astrocytes and potentially very few tumor cells mainly infiltrating along blood vessels and axonal tracts.^14,32^ Those areas were chosen because of their histological features, which could be used to provide the ground truth for the spectral classifier. For reuse, the CaF2 slides were washed after this process. To do so, the slides are placed in a bath of warm rinsing agent (consisting mainly of surface-active agents and ethanol) for 30 min and then cleaned manually, until tissue residues could no longer be seen visually. Finally, the slides are washed off with distilled water and adhering tissue residues were spectroscopically excluded.

### Raman Spectroscopy

The ProRaman-L high-performance Raman spectrometer (TSI, Shoreview, USA) (Supplementary Figure 2) has an excitation laser at 785 nm and a CCD sensor with a cooling temperature of –60°C enabling repeatable measurements.^33^ For data acquisition, a lens with 7 mm working distance and 100 μm spot size was used. The defined areas of the tumor tissue were measured repeatedly, but at different locations within the desired area on CaF_2_ slides. The acquisition parameters of the measurement were set to 10 s, 30 averages, 90 mW. The data was recorded and saved as a.spc file using the ProRaman Reader software Version 8.3.6 (TSI, Shoreview, MN).

### Data Analysis and Machine Learning

**Figure 2.**
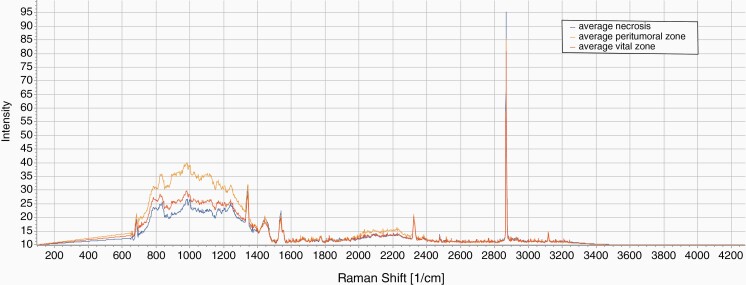
Raman spectroscopy data of histologically defined areas in GBM. Average spectra of the three histologically defined areas in GBM (vital tissue / peritumoral tissue / necrosis). For a better overview, the CaF_2_ peak at 321 cm^–1^ has been subtracted. SPECTRAGRYPH software was used for data analyses.

We divided our measurements into two data sets: one set for training a classifier (471 measurements, 100 FFPE blocks from 48 tumors) and a separate, balanced set (99 measurements, 17 FFPE blocks from 11 tumors) for testing and validating the classifier with measurements from different samples to minimize the bias of tumor sample dependent measurement identification. The training data set contained 100 measuring points of necrosis and 105 measuring points of peritumoral zone and 266 measuring points of the vital zone, whereas the second independent data set contained 29 measuring points of necrosis, 37 measuring points of peritumoral area and 33 measuring points of the vital zone. An overview on the number of measurements in the individual histological areas for both data sets is given in Table 1. We used the software SPECTRAGRYPH^34^ (Menges Friedrich, Oberstdorf, Germany) for the optical display of individual spectra as well as for processing and displaying of the average spectra of the three areas (Figure 2). Data analysis was done using the MATLAB Toolbox (MathWork, Natick, MA) for Statistics and Machine Learning™.^35^ The MATLAB Classification Learner App^36^ is an interactive and user-friendly way to practice supervized machine learning. We trained a classifier in the Classification Learner App with the different areas as response variables, the intensities of the Raman shifts as predictor variables and 5-fold cross-validation. The app trains a model for each fold and the average test error over all folds is calculated.^37^ We chose SVMs (support vector machines) as a method of pattern recognition and trained the algorithm with our data. The resulting classifier was subjected to a hyperparameter optimization; an automatic selection and improvement of hyperparameters to tune our SVM.^38^ We calculated the performance statistic based on the output of the SVM classifier. The post-test probabilities were calculated by the Classification Lerner App; the total accuracy was calculated as the percentage of correct classifications out of all classifications.

**Table 1.** Data Overview

Sample size: 117 FFPE blocks from 59 glioblastomas		
Pathohistological area (*n* = 3)	Measuring points training data set (*n* = 471; 48 tumors)	Measuring points external validation data set (*n* = 99; 11 tumors)
Necrosis	100	29
Peritumoral	105	37
Vital zone	266	33

Overview about the number of glioblastomas and measurements carried out in total as well as distribution of the measurements related to the two data sets. The external validation data set contains different tumors to avoid bias.

## Results

### Classifier Sesults Show Division Into Three Different Spectral Groups

**Figure 3.**
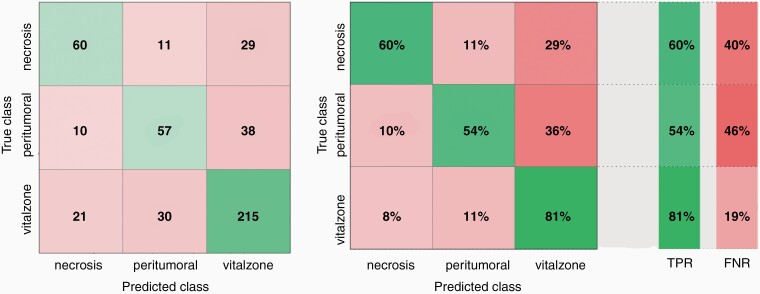
SVM of training data set after validation. Performance of our classifier after internal 5-fold cross-validation. (A) Number of observations of the established classifier, plotted according to histological area (= true class) and prediction based on the Raman spectra (= predicted class). (B) Corresponding TPR (True Positive Rate) and FNR (False Negative Rate) values. The TPR was for vital zone 81% (FNR 19%), for necrosis 60% (FNR 40%) and for peritumoral zone 54% (FNR 46%).

**Figure 4.**
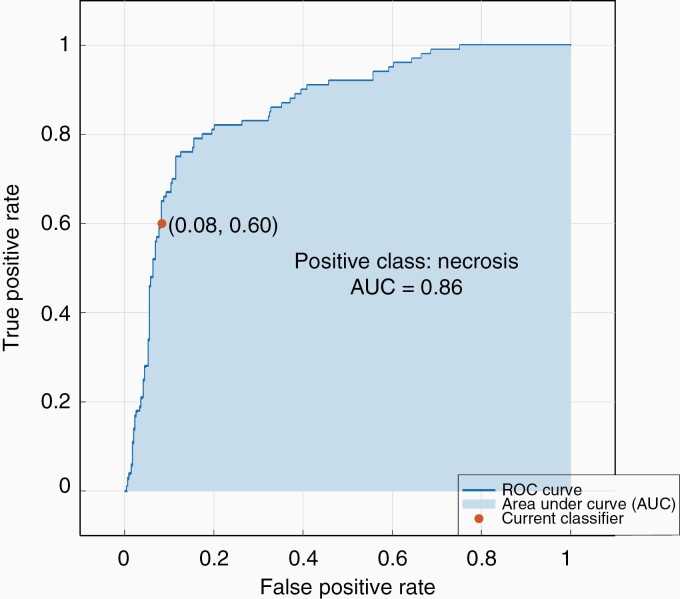
ROC curve and AUC value of our SVM. The ROC curve (receiver operating characteristics curve) and the AUC value (0.86) are shown here and plotted using the example of the necrosis zone.

We established a multiclass categorical classifier with a total accuracy of 70.5% (332 correct vs 139 misclassified) and plotted the number of observations as well as the class prediction results in a confusion matrix (Figure 3). Our classifier revealed a True Positive Rate (TPR) of 81% for the vital zone, 60% for necrosis and 54% for the peritumoral zone. The False Negative Rate (FNR) was 19% for the vital zone, 46% for the peritumoral zone and 40% for necrosis, respectively (Figure 3). We obtained a Positive Predictive Value (PPV) rate for the vital zone of 76%, for necrosis of 66%, and for the peritumoral zone 58% as well as a False Discovery Rate (FDR) for the vital zone of 24%, for necrosis of 34%, and for the peritumoral zone of 42% (Supplementary Figure 3). In addition, the ROC (receiver operating characteristics curve) curve and the AUC (area under the curve) number can be used as an indicator of the classifier performance, even for multi-class classifications.^39^ We received a ROC for each of the three classes. The corresponding AUC value for the necrosis class was 0.86 (Figure 4), for vital zone class 0.81 and for peritumoral zone class 0.80.

### Good Discrimination Between Necrosis and Peritumoral Area Even in Unbalanced Training Data

**Figure 5.**
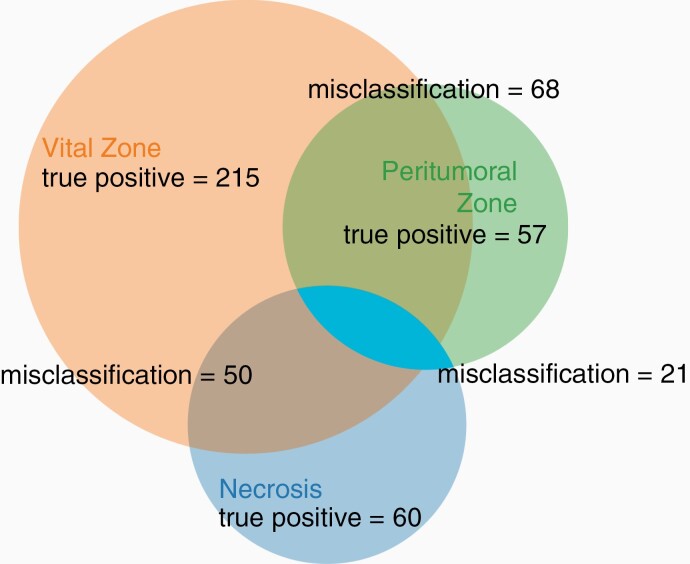
Distribution of the classifier predictions. Results for the training data set. The non-transparent turquoise blue area represents the misclassification between necrosis and peritumoral zone. On the one hand, measuring points can be divided into three different groups. On the other hand, the number of incorrect classifications between the peritumoral zone and the necrosis is visualized.

As a result of the area distribution within the tumor tissue, the number of measuring points corresponding to one of the three areas is not distributed equally (refer to Table 1). The surgeon used imaging techniques or intraoperative neuronavigation and microscopy (including labeling with aminolevulinic acid) as well as visual and tactile impressions to resect tumor tissue, which, compared to peritumoral or healthy brain tissue, is resected in larger quantities. Of note, our classifier has been trained with an unbalanced data set reflecting this distribution. The detection of the vital zone revealed the highest TPR at 81% and PPV at 76%, which is dependent on the frequency of vital zones appearing in our data set. Most misclassifications occurred between the vital zone and the necrosis / peritumoral zone, respectively, while only a small percentage (in total 21 mispredictions of 471 measurements) is between necrosis and peritumoral zone (Figure 5), suggesting a good separability of these two areas using our established classifier.

### Classifier Validation

We tested our classifier with a balanced dataset of different tumors to exclude the imbalance of input data. Testing the classifier with an extra data set is also a protection against overfitting after the optimization process.^38^ The true positive rate / true negative rate (TNR) in this classification was 59% (TPR) / 94% (TNR) for necrosis, 54% (TPR) / 94% (TNR) for peritumoral zone and 79% (TPR) / 58% (TNR) for vital zone (Supplementary Figure 4). Amongst the misclassifications, there was only one overlap (misclassification) between necrosis and peritumoral zone, all other misclassification occurred either between vital zone and necrosis (12 necrosis as vital zone, 3 vital zones as necrosis) or vital zone and peritumoral zone (16 peritumoral zones as vital zone, 4 vital zones as peritumoral zone). This mirrors the situation in the 5-fold cross-validation of the classifier training, suggesting a good separation between the peritumoral zone and necrosis. The vital zone is also a distinct spectral entity but more frequently misidentified as either of the other ones. This reflects the morphological arrangement of the zones. For a closer look at the sensitivity, specificity and positive predictive value/false discovery rate obtained from the data set for validation, see Table 2.

**Table 2.** Classifier Results of the Validation Data Set

	Necrosis	Peritumoral zone	Vital zone
True positive rate (=sensitivity)	59% (17/29)	54% (20/37)	79% (26/33)
True negative rate (=specificity)	94% (66/70)	94% (58/62)	58% (38/66)
Positive predictive value	81% (17/21)	83% (20/24)	48% (26/54)
False discovery rate	19% (4/21)	17% (4/24)	52% (28/54)

Overview of sensitivity, specificity and positive predictive value when using SVM classifier for the validation cohort (*n* = 99).

## Discussion

Our data shows that RS can distinguish histologically defined zones in GBM even upon aggressive chemical treatment leading to a considerable change of the biochemical tissue composition as in FFPE samples.

A considerable number of spectra acquired from the vital zone shows spectral similarities to either necrosis or infiltrative peritumoral areas, however, most spectra of this area are still recognized as a distinct spectral entity by the classifier. The clearest separation is achieved between the necrotic and the peritumoral areas of the tumor. This is in line with previous findings on frozen tumor samples.^40,41^ In our SVM classifier, all three histological areas are presented as distinct spectral entities. This could be confirmed with a balanced, independent data set revealing a good differentiation between necrosis and peritumoral zone. This confirms that GBM cannot be sufficiently determined by a single type of spectral property, which is an important fact to be considered for global RS-based classifiers aiming at distinguishing different tumor entities. For a global classifier approach, eg, using RS as an intraoperative diagnostic tool to identify the adequate resection margin, future studies may use our findings to distinguish the zones in fresh, unprocessed tissue as well. However, the translation of spectra across different levels of processing is challenging and will require additional analytic tools. The Raman shift values provide information about the underlying biochemical properties of the sample. However, the approach used in this study does not make use of such information but treats the spectra as fingerprint-like patterns. This leaves several potential sources of influence such as the formalin fixation or individual residues of paraffin wax sticking to the tissue, difficult to access. Despite optimization protocols, we thus cannot exclude residue spectra.

While necrosis and peritumoral zone can be well distinguished by our classifier, we see some misclassification between the vital zone and of both adjacent areas. There are several possible explanations for this phenomenon. The classifier was trained based on the histological assignment of areas as one of the three classes, however, the precision of this assignment is naturally limited. In representative tumor tissue, the three areas are in tight morphological association and the identification of the histological ground truth was not done on the measured section but on the adjacent one, which was stained. In the small deviation between these two sections, some spatial shifts may explain the observed differences between the assigned class and the one predicted by the classifier. Even within the same section, the intimate intertwining of features may contribute to a heterogenous spectral signal within the spot size of the excitation laser, which is 100 µm in diameter. It would be possible to increase the resolution with smaller spot sizes, however, we chose a spot-measurement-based method that, in contrast to microscopic Raman scanning, aims to provide a fast readout with a single measurement that is largely insensitive to sampling errors. Furthermore, it could be assumed that necrotic areas may still contain biochemical constituents that appear within the spectral range of the vital zone. As proposed in a rat model, central necrosis (no signs of proliferation) and peripheral necrosis (associated not only with pyknotic but also pseudopalisading cells) display different spectral identities, attributed to a different concentration of plasma proteins or high lipid content.^42^ Therefore our necrotic area itself may display a merging zone, of which some spectral overlap with the vital zone occurs.

Taken together, the classification of tumor areas is based on fundamentally different properties of the tissue, on the one hand, the morphological appearance, versus on the other hand the biochemical composition. Given the good discrimination between the peritumoral zone and necrosis and the identification of a distinct but overlapping spectral class for the vital zone, this tool presents a useable method for the identification of tissue even when not all required features for diagnostics are present in a sample. This taken into account, our method of measurement and classification does provide a good way to identify the different zones in an unstained section of an FFPE sample. Even if only one of the histological areas would be present in a given sample, the classifier could be used for diagnostics, in contrast to histopathological assessment, which requires all three. Further studies are needed to determine if this also holds true against similar areas of different origin, such as necrosis in a metastasis. Further studies with complementary analyzes (eg, mass spectroscopy) could offer the possibility of determining changed biochemical processes in the course of the previous fixation and paraffin wax embedding.

In conclusion, this study demonstrates the possibility of classifying different tumor areas on FFPE GBM tissue with the help of Raman spectroscopy. This may be a useful, relatively cheap and easy-to-apply tool to complement histopathological and molecular diagnostics. Like other qualitative methods, it provides an unbiased approach to tumor diagnostics with very little requirements to the sample.

## Supplementary Material

vdab077__suppl_Supplementary_Materials

## Notes

**Authorship Statement.** F.K., M.M., F.H., A.H. Study design. G.K., F.J., G.M., K.K., R.S. Sample acquisition. G.K., F.K. Machine learning. F.K., G.K., M.M. Analysis and interpretation of the results. M.M., J.G. Processing, analysis and interpretation of the neuropathological samples. F.K., M.M., A.H., F.H. Supervision and coordination of the project. G.K. Writing of the first draft. All authors contributed to the final draft.

## References

[1] Auner GW , Koya SK, Huang C, et al. Applications of Raman spectroscopy in cancer diagnosis. Cancer Metastasis Rev. 2018;37(4):691–717. 30569241 10.1007/s10555-018-9770-9 PMC6514064

[2] Anna I , Bartosz P, Lech P, Halina A. Novel strategies of Raman imaging for brain tumor research. Oncotarget. 2017;8(49):85290–85310. 29156720 10.18632/oncotarget.19668 PMC5689610

[3] Zhang J , Fan Y, He M, et al Accuracy of Raman spectroscopy in differentiating brain tumor from normal brain tissue. Oncotarget. 2017;8(22):36824–36831. 28415660 10.18632/oncotarget.15975 PMC5482701

[4] Singh R . C. V. Raman and the discovery of the raman effect. Phys Perspect. 2002;4(4):399–420.

[5] Livermore LJ , Isabelle M, Bell IM, et al. Rapid intraoperative molecular genetic classification of gliomas using Raman spectroscopy. Neurooncol Adv. 2019;1(1):vdz008. 31608327 10.1093/noajnl/vdz008 PMC6777649

[6] DePaoli D , Lemoine É, Ember K, et al. Rise of Raman spectroscopy in neurosurgery: a review. J Biomed Opt. 2020;25(5):1–36. 10.1117/1.JBO.25.5.050901 PMC7195442 32358930

[7] Huang Z , McWilliams A, Lam S, et al. Effect of formalin fixation on the near-infrared Raman spectroscopy of normal and cancerous human bronchial tissues. Int J Oncol. 2003;23(3):649–655. 12888900

[8] Draux F , Gobinet C, Sulé-Suso J, et al. Raman spectral imaging of single cancer cells: probing the impact of sample fixation methods. Anal Bioanal Chem. 2010;397(7):2727–2737. 20490470 10.1007/s00216-010-3759-8

[9] Gaifulina R , Maher AT, Kendall C, et al Label-free Raman spectroscopic imaging to extract morphological and chemical information from a formalin-fixed, paraffin- embedded rat colon tissue section. Int J Exp Pathol. 2016;97(4)::337–350. 27581376 10.1111/iep.12194 PMC5061758

[10] Fullwood LM , Clemens G, Griffiths D, et al Investigating the use of Raman and immersion Raman spectroscopy for spectral histopathology of metastatic brain cancer and primary sites of origin. Anal Methods. 2014;6(12):3948–3961.

[11] Mian S , Colley H, Thornhill M, Rehman I. Development of a dewaxing protocol for tissue-engineered models of the oral mucosa used for Raman spectroscopic analysis. Appl Spectrosc Rev. 2014;49:614–617.

[12] Faoláin EO , Hunter MB, Byrne JM, et al. Raman spectroscopic evaluation of efficacy of current paraffin wax section dewaxing agents. J Histochem Cytochem. 2005;53(1):121–129. 15637345 10.1177/002215540505300114

[13] Fullwood LM , Ashton K, Dawson T, et al Effect of substrate choice and tissue type on tissue preparation for spectral histopathology by Raman microspectroscopy. Analyst. 2014:446–454. 10.1039/c3an01832f 24308030

[14] Louis DN , Ohgaki H, Wiestler OD, et al World health organization classification of tumours of the central nervous system. In: Louis DN, Ohgaki H, Wiestler OD, Cavenee WK, eds. 4th ed. Lyon: International Agency for Research on Cancer; 2016.

[15] Wirsching HG , Galanis E, Weller M. Glioblastoma. Handb Clin Neurol. 2016;134:381–397. 26948367 10.1016/B978-0-12-802997-8.00023-2

[16] Lemée JM , Clavreul A, Menei P. Intratumoral heterogeneity in glioblastoma: don’t forget the peritumoral brain zone. Neuro Oncol. 2015;17(10):1322–1332. 26203067 10.1093/neuonc/nov119 PMC4578587

[17] D’Alessio A , Proietti G, Sica G, Scicchitano BM. Pathological and molecular features of glioblastoma and its peritumoral tissue. Cancers (Basel). 2019;11(4):469. 10.3390/cancers11040469 PMC6521241 30987226

[18] Capper D , Jones DTW, Sill M, et al DNA methylation-based classification of central nervous system tumours. Nature. 2018;555(7697):469–474. 29539639 10.1038/nature26000 PMC6093218

[19] Silantyev AS , Falzone L, Libra M, et al Current and future trends on diagnosis and prognosis of glioblastoma: from molecular biology to proteomics. Cells. 2019;8(8):863. 10.3390/cells8080863 PMC6721640 31405017

[20] Reddy SP , Britto R, Vinnakota K, et al. Novel glioblastoma markers with diagnostic and prognostic value identified through transcriptome analysis. Clin Cancer Res. 2008;14(10):2978–2987. 18483363 10.1158/1078-0432.CCR-07-4821

[21] Jovčevska I . Next generation sequencing and machine learning technologies are painting the epigenetic portrait of glioblastoma. Front Oncol. 2020;10(May):1–14. 32500035 10.3389/fonc.2020.00798 PMC7243123

[22] Deo RC . Machine learning in medicine. Circulation. 2015;132(20):1920–1930. 26572668 10.1161/CIRCULATIONAHA.115.001593 PMC5831252

[23] Qifang Bi, Goodman KE, Kaminsky J, Lessler J. What is Machine Learning? A primer for the epidemiologist. Am J Epidemiol. 2019;188(12):2222–2239. doi:10.1093/aje/kwz189. 31509183

[24] Komura D , Ishikawa S. Machine learning approaches for pathologic diagnosis. Virchows Arch. 2019;475(2):131–138. 31222375 10.1007/s00428-019-02594-w

[25] Pallua JD , Brunner A, Zelger B, Schirmer M, Haybaeck J. The future of pathology is digital. Pathol Res Pract. 2020;216(9):153040. 32825928 10.1016/j.prp.2020.153040

[26] Rashidi HH , Tran NK, Betts EV, Howell LP, Green R. Artificial intelligence and machine learning in pathology: the present landscape of supervised methods. Acad Pathol. 2019;6:2374289519873088. 31523704 10.1177/2374289519873088 PMC6727099

[27] Smith WS , Keenan KJ, Lovoi PA. A unique signature of cardiac-induced cranial forces during acute large vessel stroke and development of a predictive model. Neurocrit Care. 2020;33(1):58–63. 31591693 10.1007/s12028-019-00845-x PMC12232733

[28] Hertz AM , Hertz NM, Johnsen NV. Identifying bladder rupture following traumatic pelvic fracture: a machine learning approach. Injury. 2020;51(2):334–339. 31866131 10.1016/j.injury.2019.12.009

[29] Official Journal of the European Union. General Data Protection Regulation. 2016. https://eur-lex.europa.eu/legal-content/EN/TXT/PDF/?uri=CELEX:32016R0679. Accessed November 5, 2020.

[30] WMA – The World Medical Association. WMA Declaration of Helsinki – Ethical Principles for Medical Research Involving Human Subjects. https://www.wma.net/policies-post/wma-declaration-of-helsinki-ethical-principles-for-medical-research-involving-human-subjects/. Accessed November 9, 2020. 10.1191/0969733002ne486xx 16010903

[31] Crystran, Poole, UK. Raman Grade Calcium Fluoride. https://www.crystran.co.uk/raman-substrate-materials. Accessed November 3, 2020.

[32] Hara A , Kanayama T, Noguchi K, et al. Treatment strategies based on histological targets against invasive and resistant glioblastoma. J oncol. 2019;2019:2964783. 31320900 10.1155/2019/2964783 PMC6610731

[33] TSI, Shoreview, USA. ProRaman-L High Performance Raman Spectrometer -. https://tsi.com/discontinued-products/proraman-l-high-performance-raman-spectrometer/. Accessed November 3, 2020.

[34] Menges F . “Spectragryph - optical spectroscopy software.” 2020;(Version 1..2.14). http://www.effemm2.de/spectragryph/. Accessed November 4, 2020.

[35] MathWork, Natick, MA. Statistics and Machine Learning Toolbox Documentation - MathWorks Deutschland. https://de.mathworks.com/help/stats/index.html?s_tid=CRUX_lftnav. Accessed November 4, 2020.

[36] MathWork, Natick, MA. Classification Learner App - MATLAB & Simulink - MathWorks Deutschland. https://de.mathworks.com/help/stats/classification-learner-app.html. Accessed November 4, 2020.

[37] MathWork, Natick, MA. Select Data and Validation for Classification Problem - MATLAB & Simulink - MathWorks Deutschland. https://de.mathworks.com/help/stats/select-data-and-validation-for-classification-problem.html. Accessed November 4, 2020.

[38] MathWork, Natick, MA. Hyperparameter Optimization in Classification Learner App - MATLAB & Simulink - MathWorks Deutschland. https://de.mathworks.com/help/stats/hyperparameter-optimization-in-classification-learner-app.html. Accessed November 4, 2020.

[39] MathWork, Natick, MA. Assess Classifier Performance in Classification Learner - MATLAB & Simulink - MathWorks Deutschland. https://de.mathworks.com/help/stats/assess-classifier-performance.html. Accessed November 4, 2020.

[40] Koljenović S , Choo-Smith LP, Bakker Schut TC, Kros JM, van den Berge HJ, Puppels GJ. Discriminating vital tumor from necrotic tissue in human glioblastoma tissue samples by Raman spectroscopy. Lab Invest. 2002;82(10):1265–1277. 12379761 10.1097/01.lab.0000032545.96931.b8

[41] Kast R , Auner G, Yurgelevic S, et al. Identification of regions of normal grey matter and white matter from pathologic glioblastoma and necrosis in frozen sections using Raman imaging. J Neurooncol. 2015;125(2):287–295. 26359131 10.1007/s11060-015-1929-4

[42] Amharref N , Beljebbar A, Dukic S, et al. Discriminating healthy from tumor and necrosis tissue in rat brain tissue samples by Raman spectral imaging. Biochim Biophys Acta. 2007;1768(10):2605–2615. 17761139 10.1016/j.bbamem.2007.06.032

