# Supplementary material for: Application of Raman spectroscopy for detection of histologically distinct areas in formalin-fixed paraffin-embedded glioblastoma
Source: Neurooncol Adv. 2021 Jun 18;3(1):vdab077. doi: 10.1093/noajnl/vdab077 (PMC8331050; doi:10.1093/noajnl/vdab077)
Supplement: vdab077__suppl_Supplementary_Materials [file vdab077__suppl_supplementary_materials.docx]

**SUPPLEMENTARY MATERIAL**

**
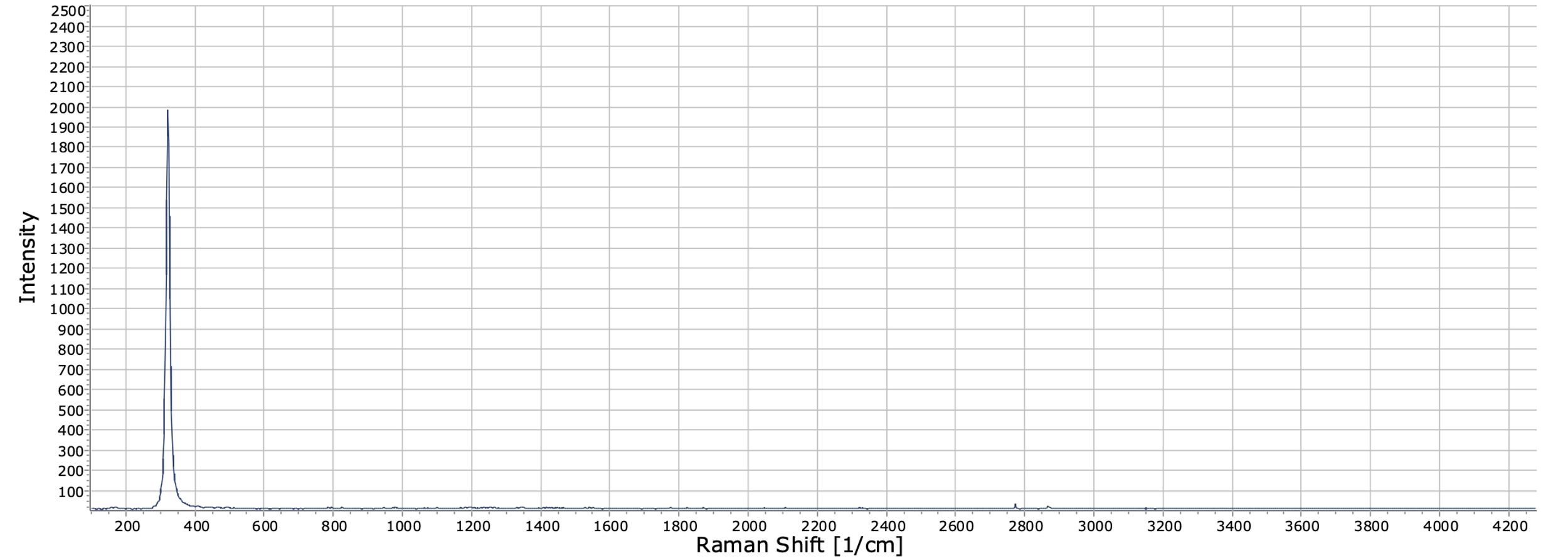
**

**Supplemental Figure 1.** **Raman spectroscopy of CaF_2_ slides.**

The spectral property of CaF_2_ slides, showing only a single peak in the Raman spectroscopy at 321 cm^-1^, allows tissue examination without broad spectral background.

**
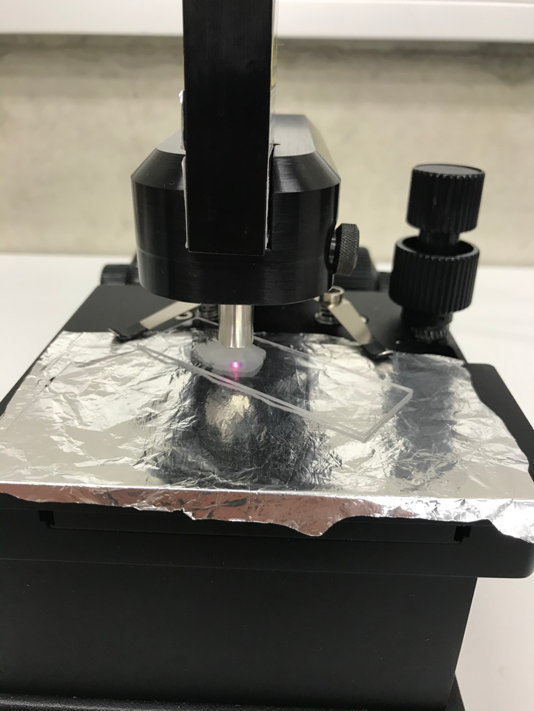
**

**Supplemental Figure 2.** **TSI ProRaman-L high-performance Raman spectrometer set up.**

Excitation laser and a slide with unstained tissue are depicted


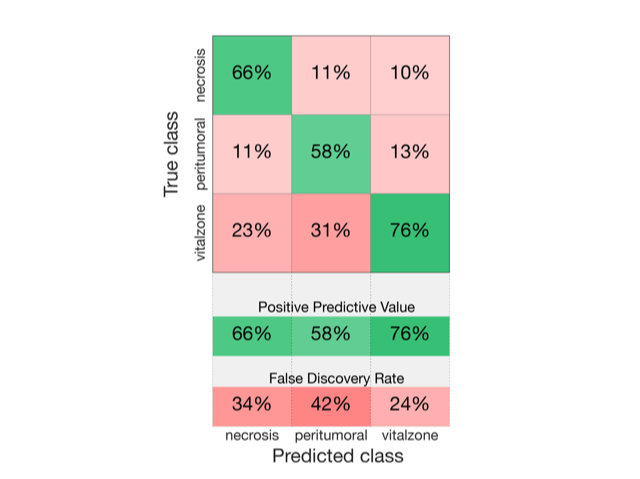


**Supplemental Figure 3.** **Confusion matrix of the** **Positive Predictive Value of our classifier.**

Confusion matrix displaying the PPV (Positive Predictive Value) and the FDR (False Discover Rate) of our classifier.


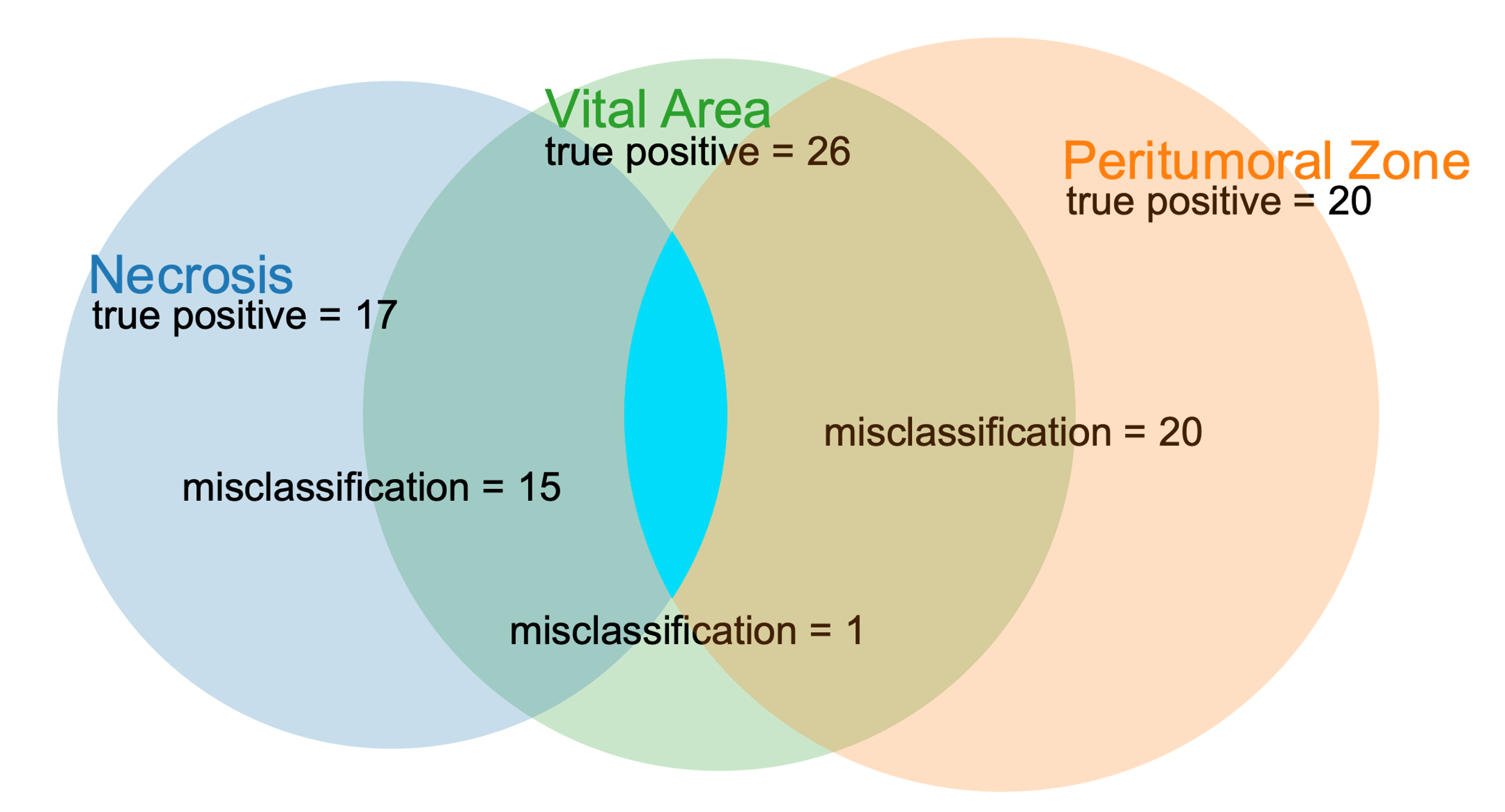


**Supplemental Figure 4.** **A Venn diagram showing the classifier performance on an external validation set.**

The proportional Venn diagram shows the performance of our classifier using the example of our extra data set (n = 99). Only one false prediction (misclassification) between necrosis and peritumoral zone was obtained.

| Tumor sample no° | Age | Sex | Localization | IDH status (wildtype / mutant) | MGMT status (methylated / unmethylated) | Primary / secondary / recurrence tumor |
| --- | --- | --- | --- | --- | --- | --- |
| 1 | 74 | female | right postcentral | wildtype | unmethylated | primary |
| 2 | 44 | female | left temporal | wildtype | methylated | primary |
| 3 | 50 | male | right temporal | wildtype | unmethylated | primary |
| 4 | 70 | male | left temporo-dorsal | wildtype | unmethylated | primary |
| 5 | 61 | male | parieto-occipital | wildtype | methylated | primary |
| 6 | 66 | male | left frontal | wildtype | methylated | primary |
| 7 | 66 | male | left temporal | wildtype | unmethylated | primary |
| 8 | 38 | male | right temporal | wildtype | not specified | recurrence |
| 9 | 51 | male | not specified | wildtype | not specified | recurrence |
| 10 | 45 | female | right temporal | wildtype | unmethylated | recurrence |
| 11 | 61 | male | right fronto-temporal | wildtype | methylated | primary |
| 12 | 50 | female | bifrontal | wildtype | methylated | primary |
| 13 | 62 | male | left temporal | wildtype | unmethylated | primary |
| 14 | 61 | male | right fronto-temporal | wildtype | methylated | primary |
| 15 | 45 | female | right frontal | not specified | unmethylated | primary |
| 16 | 54 | female | left occipital | wildtype | unmethylated | primary |
| 17 | 39 | female | right frontal | wildtype | methylated | primary |
| 18 | 58 | not specified | right frontal | wildtype | methylated | primary |
| 19 | 56 | not specified | left temporal | not specified | methylated | primary |
| 20 | 62 | male | left temporal | wildtype | unmethylated | primary (resection) |
| 21 | 48 | male | right parietal | wildtype | unmethylated | primary |
| 22 | 64 | female | right occipital | wildtype | methylated | primary |
| 23 | 59 | female | right parieto-occipital | not specified | not specified | primary |
| 24 | 67 | male | left fronto-basal | wildtype | unmethylated | primary |
| 25 | 51 | male | right temporal | wildtype | methylated | primary |
| 26 | 69 | female | multifocal | wildtype | methylated | primary |
| 27 | 72 | female | right temporal | wildtype | methylated | primary |
| 28 | 61 | male | fronto-parietal | wildtype | methylated | primary |
| 29 | 84 | female | right occipital | not specified | unmethylated | primary |
| 30 | 61 | male | right temporal | wildtype | methylated | recurrence |
| 31 | 55 | female | left occipital | wildtype | unmethylated | recurrence |
| 32 | 67 | male | left temporal | wildtype | not specified | primary |
| 33 | 62 | male | right frontal | wildtype | unmethylated | primary |
| 34 | 72 | male | right opercular | wildtype | methylated | primary |
| 35 | 64 | male | right fronto-dorsal | wildtype | methylated | primary |
| 36 | 69 | male | left fronto-parietal | wildtype | unmethylated | primary |
| 37 | 50 | female | left temporal | wildtype | methylated | primary |
| 38 | 64 | male | left temporal and parietal | wildtype | methylated | primary |
| 39 | 67 | male | right parietal | wildtype | methylated | recurrence |
| 40 | 57 | male | right frontal | wildtype | unmethylated | primary |
| 41 | 42 | female | right temporal | mutant | not specified | secondary |
| 42 | 75 | female | right central | wildtype | unmethylated | secondary |
| 43 | 75 | female | not specified | wildtype | unmethylated | recurrence |
| 44 | 75 | female | right parietal | wildtype | unmethylated | recurrence |
| 45 | 80 | male | left frontal | wildtype | methylated | not specified |
| 46 | 56 | male | left occipital | wildtype | not specified | primary |
| 47 | 56 | male | not specified | not specified | unmethylated | recurrence |
| 48 | 81 | male | left temporal | wildtype | methylated | primary |
| 49 | 44 | female | right frontal | wildtype | unmethylated | primary |
| 50 | 44 | male | left temporo-mesial | wildtype | unmethylated | recurrence |
| 51 | 66 | male | parietal | wildtype | not specified | primary |
| 52 | 58 | male | left occipital | wildtype | unmethylated | primary |
| 53 | 53 | male | Corpus callosum | wildtype | unmethylated | primary |
| 54 | 51 | male | right frontal | wildtype | unmethylated | primary |
| 55 | 60 | male | left temporal | wildtype | unmethylated | primary |
| 56 | 78 | male | right temporal | wildtype | methylated | primary |
| 57 | 82 | male | temporo-insular | wildtype | unmethylated | primary |
| 58 | 54 | male | left temporal | wildtype | unmethylated | primary |
| 59 | 54 | male | right frontal | wildtype | unmethylated | primary |

**Supplementary Table 1.**

Description and more detailed information of the examined glioblastoma samples.
